# Relationship Between Vitamin D and Thyroid: An Enigma

**DOI:** 10.7759/cureus.21069

**Published:** 2022-01-10

**Authors:** Tejaswini Ashok, Vishnu Palyam, Ahmad T Azam, Oladipo Odeyinka, Rasha Alhashimi, Sankeerth Thoota, Ibrahim Sange

**Affiliations:** 1 Internal Medicine, Jagadguru Sri Shivarathreeshwara (JSS) Medical College, Mysore, IND; 2 Internal Medicine, Jagadguru Jayadeva Murugarajendra (J.J.M) Medical College, Davanagere, IND; 3 Internal Medicine, Allama Iqbal Medical College, Lahore, PAK; 4 Internal Medicine, University of Ibadan College of Medicine, Ibadan, NGA; 5 Internal Medicine, University of Baghdad College of Medicine, Baghdad, IRQ; 6 Internal Medicine, Meenakshi Medical College Hospital and Research Institute, Kancheepuram, IND; 7 Research, K.J. Somaiya Medical College, Hospital and Research Centre, Mumbai, IND

**Keywords:** anti-tpo antibodies, low vitamin d, vitamin d supplementation, papillary carcinoma of thyroid, hypothyroidism, autoimmune thyroid disorders, vitamin-d

## Abstract

Hypothyroidism is a frequently encountered endocrine disorder in clinical practice. Besides its traditional role in bone health, vitamin D has been shown to have favorable effects in a variety of different systems due to its pleiotropic qualities and ubiquitous receptor expression. Over the years, researchers have been fascinated by the intricate molecular interplay between vitamin D and thyroid. In this regard, attempts have emerged to demonstrate the role of vitamin D in thyroid disorders. This article has reviewed the existing literature on the role of vitamin D in hypothyroidism. We explored studies discussing the physiological interactions between vitamin D and thyroid, as well as the clinical consequences, supplemental and prognostic relevancy of vitamin D in auto-immune thyroid disease (AITD) and hypothyroidism.

## Introduction and background

Hypothyroidism is a common pathological disease of thyroid hormone deficiency, with a prevalence in the general population between 0·3% and 3·7% in the United States and between 0·2% and 5·3% in Europe [1-5]. Undiagnosed hypothyroidism, including both overt and mild instances, was estimated to be roughly 5% in meta-analysis research done across nine European countries [2]. Various epidemiological studies have indicated that women have a higher predisposition to developing hypothyroidism than men [6]. Iodine deficiency continues to be the leading cause of hypothyroidism worldwide, while populations in iodine sufficient areas suffer from hypothyroidism due to autoimmune (Hashimoto's thyroiditis) and iatrogenic causes [7]. The wide spectrum of symptoms associated with hypothyroidism implies that it has an influence on metabolism and multiple organ systems [7]. The typical clinical symptoms encountered in outpatient practice are fatigue, cold intolerance, dry skin, constipation, muscle aches, decreased sweating, thickening of the skin, brittle hair, hair loss, loss of the lateral eyebrows, etc. [7].

Clinical/overt hypothyroidism is defined by an increased thyroid-stimulating hormone (TSH) and low free thyroxine (FT4) levels, whereas subclinical hypothyroidism is characterized by normal FT4 levels but an elevated serum TSH, provided the hypothalamic-pituitary-thyroid axis is intact, and this pattern can be replicated for at least four weeks [7]. Daily oral administration of levothyroxine (LT4) is considered to be the gold standard of treatment for hypothyroidism because of its extended serum half-life, efficacy in resolving symptoms, acceptable side effect profile, and low cost [8]. The pivotal role of vitamin D, a fat-soluble vitamin, in calcium and phosphate homeostasis is well recognized [9]. The emerging prevalence of hypovitaminosis D in populations with hypothyroidism might be attributed to the evolution of the vitamin D3 receptor and the receptor for thyroid hormone from a single primordial gene causing a strong homology between these receptors [10,11]. Synthesis of 1,25 dihydroxy-vitamin D or calcitriol, the active metabolite of vitamin D, requires the enzyme 1-alpha hydroxylase, which is expressed predominantly in the kidney [12]. Despite evidence of a relationship between the two, the significance of vitamin D in the pathophysiology of hypothyroidism remains unknown. This review article aims to explore the molecular and clinical relationship between vitamin D levels and hypothyroidism and underline the prognostic alterations due to vitamin D supplementation in hypothyroidism.

## Review

Sources and physiology of vitamin D

Even in countries where biofortification is practiced, sunlight exposure remains the primary source of vitamin D for the majority of the population [13]. The limited natural dietary sources include oily fish such as salmon, mackerel, tuna, sardine, egg yolk, and shiitake mushrooms [14]. Cod liver oil has been considered an excellent source of vitamin D3 over centuries [15]. A few examples of vitamin D fortification include milk (100 IU per 8-ounce serving), orange juice (100 IU per 8-ounce serving), and some bread and cereals [16,17]. Increased dietary and supplemental vitamin D intakes, as well as moderate sun exposure (usually 5-10 minutes of exposure of the arms and legs or the hands, arms, and face, two or three times per week), are feasible methods of assuring vitamin D sufficiency [15].

When 7-dehydrocholesterol (7-DHC) in the skin is exposed to solar ultraviolet B photons (290 -315 nm), it is metabolized into previtamin D3 [13,15,18]. The epidermis contains around 65% of 7-DHC, and more than 95% of the previtamin D3 generated is in the viable epidermis, so it cannot be eliminated when the skin is washed [19]. The newly formed previtamin D3, which is confined within the lipid bilayer of the plasma membrane, undergoes rapid rearrangement of its double bonds to produce the more thermodynamically stable vitamin D3 [15].

**Figure 1:**
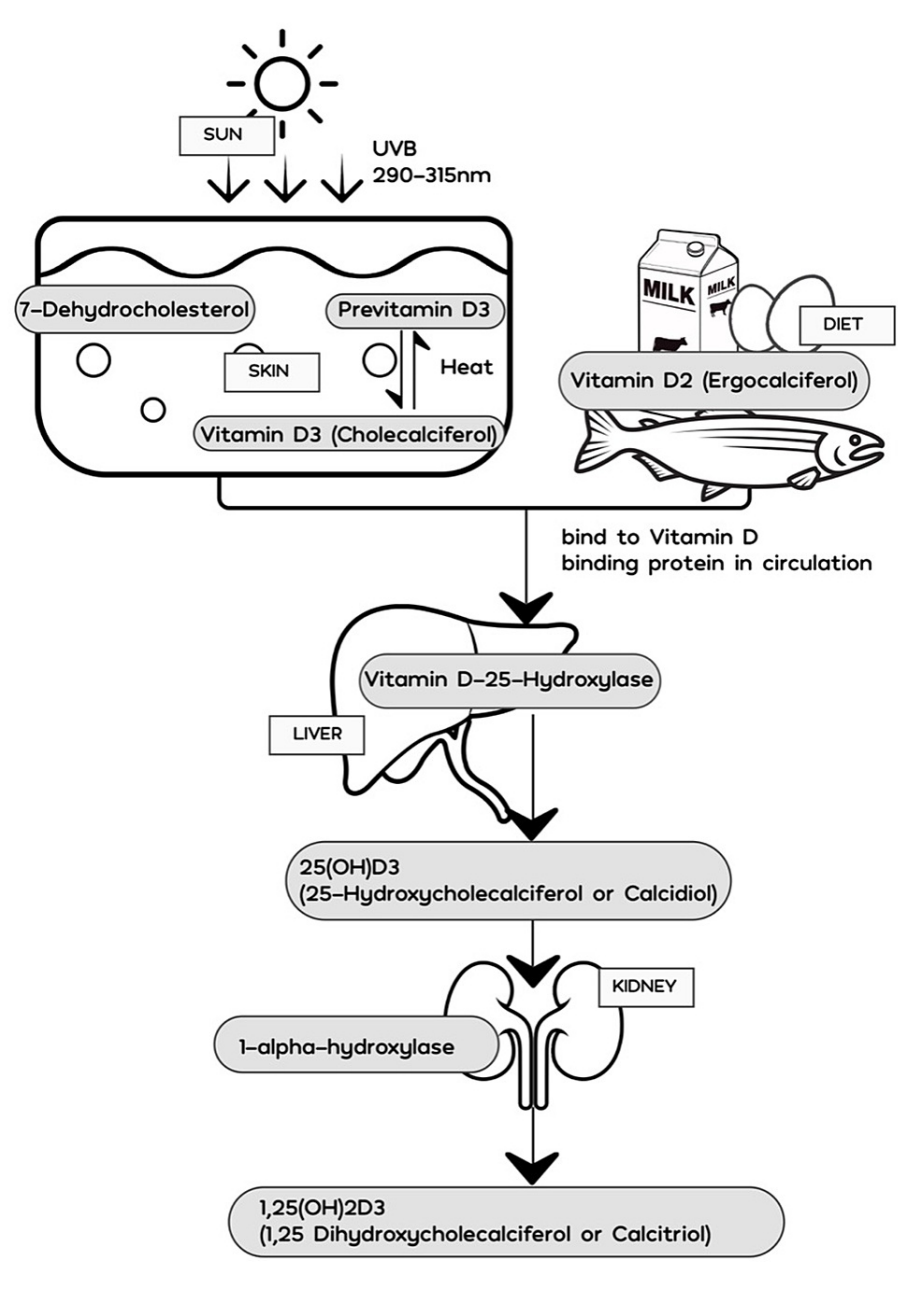
Metabolism of vitamin D

Vitamin D3 is discharged into the extracellular space during this transition process, and it is drawn into the circulation by the vitamin D-binding protein (DBP) in the dermal capillary bed [15]. The cutaneous synthesis of vitamin D3 is influenced by season, latitude, ozone levels, time of day, skin pigmentation, age, and sunscreen use [15]. Ingested forms of vitamin D are integrated into chylomicrons, which go via the lymphatic system into the venous bloodstream, where it binds to DBP and is transported to the liver [18,20]. In the hepatic parenchyma, the enzyme vitamin D-25-hydroxylase metabolizes vitamin D2 and vitamin D3 into the primary circulating vitamin D metabolite, 25-hydroxyvitamin D (25(OH)D). It is further hydroxylated in the kidneys by the enzyme 25-hydroxyvitamin D 1-alpha-hydroxylase or simply 1-alpha-hydroxylase (cytochrome P450 family 27 subfamily B member 1 or CYP27B1) to generate 1,25-dihydroxyvitamin D (1,25[OH]2D3) or calcitriol (Figure 1). The functioning of vitamin D is through the binding of calcitriol to the vitamin D receptor (VDR), which is expressed in the nucleus of all non-dividing cells. Calcitriol interacts with VDR in the small intestine to increase the efficiency of intestinal calcium and phosphorous absorption by approximately 20% [18]. Similarly, 1,25-dihydroxyvitamin D also exerts its effect on bone metabolism through its interaction with VDR in the osteoblasts, triggering a receptor activator of nuclear factor-kB ligand in osteoblasts, which then interacts with a receptor activator of nuclear factor-kB on immature preosteoclasts, causing them to develop into mature bone-resorbing osteoclasts. Through bone resorption, vitamin D helps maintain calcium and phosphorous homeostasis in the blood [21]. The ubiquitous expression of VDR draws attention to the many pleiotropic effects of vitamin D like the stimulation of insulin production, regulation of activated B and T lymphocyte activity, effects on cardiac contractility, etc. [12,22-24]. 1,25(OH)2D3 regulates the expression of about 200 genes, including those involved in the regulation of cellular proliferation, differentiation, apoptosis, and angiogenesis [18].

The presence of direct molecular interactions between vitamin D and thyroid is still poorly understood. Pioneering research from the 1980s showed that vitamin D might have a role in thyroid illness. While it was well-established that 1,25(OH)2D3 substantially increases serum calcium and phosphorus concentrations, Sowers et al., through a rodent study in 1980, demonstrated that hypercalcemia suppresses TSH and thyrotropin-releasing hormone (TRH) production via dopaminergic inhibition [25]. Further, in 1987, Tornquist and Lamberg-Allardt discovered in vivo with rats and in vitro with isolated rat pituitary cells that 1,25(OH)2D3 had no effect on baseline TSH production [26]. McDonnell et al., in 1988, discovered a strong homology between the molecular structure of the vitamin D3 receptor and the thyroid hormone receptor, which was due to two regions that they share: a 70 amino acid, cysteine-rich sequence, and a 62 amino acid sequence located near the proteins' carboxyl terminus [11]. A study published in the early 1990s discovered that 1,25(OH)2D3 suppressed TSH-stimulated iodide absorption in a dose-dependent manner, indicating that 1,25(OH)2D3 had an effect on the physiological function of rat thyroid follicular cells (FRTL-5) [27]. When FRTL-5 cells were incubated with 1,25(OH)2D3 or cholecalciferol analogs, Berg et al. found a reduction in TSH-stimulated synthesis of the intracellular signaling molecule 3′,5′-cyclic adenosine monophosphate (cAMP), iodide uptake, and cell proliferation [28]. In another study, rats were fed a significant vitamin D deficient diet, and it was demonstrated that they had reduced blood TSH levels but FT4 levels comparable to vitamin D adequate animals [29]. Furthermore, VDR knockout animals revealed only minimal changes in the anatomy and physiology of the thyroid gland and a modest decrease in circulating TSH levels [30]. These interesting findings established the groundwork for and inspired ongoing research on vitamin D and thyroid illness. Wang et al. observed a substantial connection between *VDR* gene polymorphisms and autoimmune thyroid diseases in several ethnic groups in a meta-analysis [31]. Similarly, in a sample of Turkish patients, Yazici et al. discovered that *VDR* gene TaqI TT and FokI FF genotypes were related to higher risk while BbAaTtFf genotypes were protective of Hashimoto's thyroiditis (HT) [32]. This finding, in conjunction with the use of calcitonin as a sensitive diagnostic for medullary thyroid cancer (MTC), may imply that 1,25(OH)2D-VDR signaling plays a role in the control of thyrocyte and C cell homeostasis, but further research is needed to back this up [33].

Polymorphisms in the CYP27B1 gene, which encodes the 1-alpha hydroxylase enzyme responsible for converting 25(OH)D to active 1,25(OH)2D3, have been associated with autoimmune thyroid disease (AITD), corroborating the theory that vitamin D and AITD are connected [34]. Wang et al. demonstrated that by increasing Bcl-2 expression, 1,25(OH)2D3 protects human thyrocytes against programmed cell death [35]. Vitamin D exerts a modulatory effect on the immune system by enhancing the innate immune system and suppressing the adaptive immune system [36]. VDR and 1-alpha hydroxylase are expressed by the majority of immune cells, including T cells, B cells, and antigen-presenting cells (APCs), such as dendritic cells (DCs) and macrophages. By modulating DCs and directly targeting T cells, 1,25(OH)2D3 promotes the formation of regulatory T cells (Tregs), preventing T helper type 1 (Th1) cell development [37]. 1,25(OH)2D suppresses B cell growth and differentiation into plasma cells, IgG and IgM production, memory B cell formation, and induces B cell apoptosis [37]. The immunomodulatory impact of 1,25(OH)2D3 on the thyroid is mediated by reduced expression of major histocompatibility complex (MHC) class II molecules on thyrocytes, impaired Th1 mediated inflammatory responses in thyrocytes, and the shift of T cell response towards the T helper type 2 (Th2) cell phenotype [38]. In any case, the effect of vitamin D on the thyroid warrants special investigation since it may aid in the better management of thyroid disorders.

Clinical significance of vitamin D in thyroid diseases

Many studies hint at a function for vitamin D in the development of autoimmune thyroid disorders (AITD), which has been confirmed in animal models. Mice were treated intraperitoneally with or without calcitriol (0.1-0.2 micrograms per kg body weight daily) after being previously sensitized with porcine thyroglobulin. When compared to placebo-treated mice, animals receiving inadequate vitamin D dosages showed a reduction in the degree of thyroid inflammation [39]. In CBA mice, combining 1,25(OH)2D3 with cyclosporine prevented the formation of experimental autoimmune thyroiditis (EAT) with a synergistic effect [40]. Another study found that 1,25(OH)2D3 can alleviate pathogenic alterations in the thyroid gland and repair cytokine imbalances in rats with EAT [41].

In an Indian population consisting of students, teachers, and staff aged 16-60 years, Goswami et al. conducted a community-based survey. Serum FT4, TSH, anti-thyroid peroxidase (anti-TPO) antibody, intact parathyroid hormone, 25(OH)D were measured. When the association between 25(OH)D and anti-TPO antibody levels were examined with and without correcting for age, it revealed a significant inverse correlation (r - 0.08, P = 0.04) when corrected for age [42]. The findings of this investigation might be comparable to those of another study done in Thailand by Chailurkit et al., who reported that high vitamin D status in younger adults is related to low circulating TSH [43]. These results prompted Zhang et al. to investigate the association between vitamin D and serum TSH levels irrespective of thyroid hormone levels in middle-aged and elderly people with negative thyroid autoimmunity. The authors concluded that high 1,25(OH)2D3 levels were associated with low circulating TSH levels independent of free triiodothyronine (FT3) and thyroxine. Women, on the other contrary, did not exhibit this tendency [44]. A longitudinal study of a Dutch female population found no negative association between anti-TPO antibody synthesis de novo and 1,25(OH)2D3 levels.

The researchers also compared patients with a genetic susceptibility to AITD to healthy subjects and found that 1,25(OH)2D3 levels in both groups were not lower in patients than in controls, indicating that low vitamin D levels are not associated with thyroid autoimmunity in the early stages of the disease [45]. Kivity et al. discovered that anti-thyroid antibodies were more consistently elevated in individuals with vitamin D deficiency. The prevalence of vitamin D deficiency was comparable in hypothyroid individuals with AITDs and those without, making it difficult to rule out the possibility that vitamin D deficiency is caused by hypothyroidism and not a primary factor in AITD pathogenesis [46]. This finding was consistent with the observations of Shin et al., who concluded that the 1,25(OH)2D3 level is an independent factor influencing the presence of anti-thyroid peroxidase (anti-TPO) antibodies in AITDs and that the direct causal influence of vitamin D deficiency on AITDs remains unclear [47]. Tamer et al. reported that 1,25(OH)2D3 deficiency (<30 ng/ml) was more prevalent in people with HT than in controls. Vitamin D deficiency was found to be more common in patients with overt hypothyroidism (47/50, 94%) or subclinical hypothyroidism (44/45, 98%) than in those with euthyroidism (57/66, 86%) [48]. Bozkurt et al. evaluated serum 25(OH)D status in three groups of subjects: 180 euthyroid patients with HT on a stable dose of levothyroxine, 180 euthyroid subjects with newly diagnosed HT, and 180 healthy volunteers. Severe vitamin D deficiency (<10ng/ml) was demonstrated in 48.3% of euthyroid HT, 35% of newly diagnosed HT, and 20.5% of healthy controls. Serum 25(OH)D levels were shown to be positively associated to thyroid volume (r = 0.145, P.001) and negatively associated to anti-TPO (r = -0.361, P.001) and anti-thyroglobulin (anti-Tg) antibody (r = -0.335, P.001) levels. These results suggested that vitamin D may have a role in the development of HT and/or its progression to hypothyroidism [49].

Choi et al. investigated the regulation of thyroid autoimmunity by vitamin D via estrogens in 6685 patients who received regular health checks and reported substantially lower serum 1,25(OH)2D3 levels in pre-menopausal women with AITD but not in postmenopausal women [50]. Furthermore, a notable association between low levels of 1,25(OH)2D3 and increased thyroid volume was found in female patients with newly diagnosed Grave’s disease (GD), indicating that vitamin D status may be implicated in the disease's development [51]. When Unal et al. compared newly diagnosed AITD patients to healthy age-matched controls, they inferred that both HT and GD patients had lower circulating 25(OH)D levels than controls [52]. The administration of 1,25(OH)2D3 to animal models of autoimmune diseases consistently delivered selective immunosuppressive effects capable of preventing or markedly suppressing a variety of experimental autoimmune diseases [53]. In a cross-sectional study, a total of 140 consecutive cases (70 patients with newly diagnosed GD and 70 patients with newly diagnosed HT) and 70 healthy controls were recruited, and serum TSH, FT3, FT4, anti-TPO, and anti-Tg levels were measured. After adjusting for age, TSH, and all thyroid autoantibodies, it was found that a lower serum 25(OH)D level was related to an increased risk of GD (OR = 1.09, 95% CI 1.03-1.15, P = 0.001). The same relationship was seen for HT (OR = 1.08, 95% CI 1.04-1.12, P = 0.001).

According to multivariate logistic regression analysis, every 5 nmol/L drop in serum 25(OH)D concentration was linked to a 1.55-fold (95% CI 1.18-2.02) rise in GD risk and a 1.62-fold (95% CI 1.30-2.05) decrease in HT risk [54]. Ke et al. investigated 175 AITD patients, including 51 with GD, 61 with mild HT, 63 with treated HT, and 51 controls. Mild and treated HT patients showed relatively low vitamin D levels, whereas GD patients had serum 25(OH)D levels comparable to healthy controls. Also, the researchers discovered no association between serum 25(OH)D levels and thyroid function, anti-thyroid antibodies, or serum cytokines IL-4, IL-17, or TNF-alpha in AITD patients [55].

There have been few studies demonstrating a link between vitamin D and thyroid disorders in the pediatric population. Camurdan et al. attempted to address the relationship between vitamin D levels and HT in children in one such research. When compared to sex- and age-matched controls, children with newly diagnosed HT had lower vitamin D levels [56]. Low serum vitamin D levels were shown to be substantially interconnected with AITD in a cohort of 56 Egyptian children with AITD and 56 healthy controls. The authors concluded, however, that vitamin D levels are not an independent risk factor for the progression of AITD to overt hypothyroidism and hypothesized that BMI might have an effect on serum 1,25(OH)2D3 levels [57]. Sonmezgoz et al. analyzed vitamin D levels in 68 children with HT aged 12 ± 4 years (39 females) and 68 healthy children aged 10 ± 4 years (37 females) and reported that a higher prevalence of vitamin D deficiency in HT patients (76%) than controls (35%) (P < 0.001) [58]. Evliyaoglu et al. produced supporting evidence by measuring serum 25(OH)D levels in 169 subjects and reporting that values less than 20 ng/mL were connected to HT in children and adolescents [59].

Racataianu et al. came to the conclusion that there is a bidirectional link between vitamin D and systemic inflammation in obese people, which potentially influences the severity and frequency of HT [60]. The findings of Yasmeh et al. call into question the majority of the published data, suggesting higher levels of 25(OH)D in women with HT compared to controls. No such difference was observed in males [61]. Some researchers argue that the data pointing to Vitamin D's significance in AITD is an effect of the illness rather than a cause of it. Bizzaro et al. emphasized the possibility of vitamin D deficiency as a result of AITD-related incapacity, decreased sunlight exposure, malabsorption, and corticosteroid use [62]. Increased body fat mass as a result of hypothyroidism, as well as other co-morbid autoimmune illnesses, may lead to vitamin D insufficiency in HT [63]. The occurrence of AITD coexisting with other autoimmune illnesses, such as celiac disease, has to be acknowledged as well. Celiac disease causes malabsorption and nutritional deficiencies, notably vitamin D deficiency, and has been related to an increased risk of developing other autoimmune diseases [64,65]. The impact of a gluten-free diet in HT patients was investigated by Krysiak et al.in 2018. Gluten-free vs. gluten-containing diets were given to HT patients with positive transglutaminase antibodies but no symptoms of celiac disease. The former group alone experienced a decrease in antibody titers and an increase in vitamin D levels [66]. An alternative theory is that low vitamin D levels are caused by chronic infection with intracellular bacteria that disrupt vitamin D metabolism by inducing VDR dysfunction inside phagocytes. This theory is justified by the growing evidence that AITD can be reversed by progressively restoring VDR function with VDR agonist olmesartan and subinhibitory dosages of certain bacteriostatic antibiotics [67].

Vitamin D and Cancer

Various epidemiological studies have reported an inverse relationship between vitamin D levels and the risk of acquiring cancer [68]. In a murine model of metastatic follicular thyroid cancer, Dackiw et al. demonstrated that in vivo calcitriol administration can eﬀectively restore p27 accumulation in thyroid carcinoma cells, an important tumor suppressor protein [69]. In vitro, Zabel et al. found that calcitriol and two analogs (PRI-1906 and PRI-2191) inhibited the proliferation of thyroid medullary carcinoma [70]. Another study, on the other hand, found that calcitriol increased TT cell (cell line originating from human thyroid medullary carcinoma) proliferation by increasing the expression of proliferation-associated proteins and suppressing apoptosis [71]. Following therapy with 1,25(OH)2D3, the weight and volume of xenografted thyroid cancer cells in severe combined immunodeficient mice were decreased by 50% and 22%, respectively. They also observed that cells down-regulated for fibronectin demonstrated a more aggressive growth pattern and were relatively insensitive to treatment suggesting a role for fibronectin in mediating vitamin D actions on neoplastic cell growth [72]. Khadzkou et al. demonstrated the expression of VDR and 1-alpha hydroxylase in papillary thyroid carcinoma (PTC) and normal thyroid tissue suggesting the endogenous production of calcitriol in the thyroid gland. In addition, lower VDR expression in PTC metastatic lymph nodes compared to primary PTC and normal thyroid tissue implied the possible role of vitamin D via its effects, such as inhibition of cell proliferation and promotion of cell differentiation [73].

Izkhakov et al. demonstrated that 1,25(OH)2D3 regulates the expression of extracellular matrix protein 1 (ECM1) and type II transmembrane serine protease 4 (TMPRSS4), two independent diagnostic markers of thyroid cancer [74]. Clinckspoor et al. observed an increased expression of VDR, cytochrome P450 family 24 subfamily A member 1 (CYP24A1), and CYP27B1 genes, all of which are implicated in local vitamin D signaling, in benign and differentiated malignant thyroid cancers. The expression of these genes was found to be decreased in local nodal and distant metastases, highlighting a local antitumor response to vitamin D in the early stages of cancer [75]. The relevance of vitamin D in cancer has also been recognized by the production of vitamin D analogs such as MART-10. In undifferentiated thyroid carcinoma cells, MART-10 was shown to be more potent than 1,25(OH)2D3 at suppressing tumorigenesis and metastasis. This research presents an opportunity for future in vivo trials of MART-10 application against anaplastic thyroid carcinoma [76]. Somjen et al. proposed that vitamin D has a function in tumor cell growth by regulating estrogen receptor (ER) expression. The researchers showed that a vitamin D analog JK 1624 F2-2 could modulate not only the VDR receptor and 1,25(OH)2D3 but also the ER, which is implicated in estrogen-induced cell proliferation in thyroid cancer cell lines in an ER-type specific way [77]. On investigating the effects of 1,25(OH)2D3 and its superagonistic analog CD578 on different cell lines of thyroid cancer, there was a notable impact on growth arrest, as well as a reduction in absolute cell counts in all cell lines. Even with a 1000-fold lower dosage of analog CD578, growth inhibition was observed [78]. Suzuki et al. established the efficiency of 22-oxacalcitriol, a vitamin D3 derivative, in reducing tumoral cell proliferation in a thyroid anaplastic carcinoma cell line [79]. Liu et al. showed that 1,25(OH)2D3 and its noncalciomimetic analog EB1089 inhibited thyroid cancer cell proliferation in a dose-dependent manner. This study also discovered that 1,25(OH)2D3 administration boosted the expression of p27, thus assisting in improved cellular differentiation, reduced tumor burden, and prevention of metastatic growth [80]. Bennett et al. verified this antiproliferative potential by observing cell growth suppression by 25(OH)D or 1,25(OH)2D3 in malignant (papillary, follicular, and anaplastic carcinoma) and simian virus 40-immortalized follicular cell lines [81].

Few human observational studies have yielded inconsistent data demonstrating a relationship between serum vitamin D levels and thyroid cancer. In a retrospective cohort study from Canada in 2010 by Roskies et al. on 212 patients undergoing thyroidectomy, preoperative 25(OH)D levels were recorded, and patients were stratified based on vitamin D status as vitamin D deficiency (VDD) or vitamin D sufficiency (VDS). When comparing the VDS and VDD groups, the malignancy rate jumps from 37.5% to 75%, equating to a relative risk of 2.0 (p =.03, 95% CI 1.07-2.66). This prompted the authors to speculate that vitamin D deficiency might be the first modifiable risk factor for thyroid cancer [82]. In a Turkish study by Sahin et al., low serum vitamin D was reported in 71% with papillary thyroid carcinomas, but only in 59% of controls. After regression analysis, the authors drew an association between tumour diameter and log-vitamin D3 concentrations (B = 0.207; p = 0.04) [83].

Kim et al. evaluated a total of 548 female patients who underwent total thyroidectomy for PTC and categorized them into four quartiles based on the preoperative serum 25(OH)D levels. On retrospective analysis of clinicopathologic features, it was observed that the preoperative 25(OH)D levels were significantly lower in patients with a tumor size of more than 1 cm (p = 0.041) or lymph node metastasis (p = 0.043) [84]. Stepien et al. discovered substantially decreased 1,25(OH)2D3 levels in 27 papillary cancer patients, 16 follicular cancer patients, and seven anaplastic patients when compared to 26 healthy controls [85]. Similarly, serum calcitriol levels were shown to be considerably lower in 172 differentiated thyroid cancer (DTC) patients compared to 321 healthy controls by Penna-Martinez et al. [86]. The role of the vitamin D system in the pathogenesis of DTC was confirmed by the same authors, who examined vitamin D levels and the expression of genes related to vitamin D enzymes in 253 DTC patients and 302 controls, discovering a link between DTC and low 25(OH)D and 1,25(OH)2D3 levels in certain CYP24A1 haplotypes [87].

Laney et al. reported in pilot research that vitamin D deficiency rates were comparable in individuals with thyroid nodules, thyroid cancer in remission, and active thyroid cancer. The only risk factor shown to be independently related to vitamin D deficiency was BMI >30 kg/m2 [88]. Similarly, Lizis-Kolus et al. found no difference in 25(OH)D concentrations between patients with papillary thyroid carcinoma and patients with HT [89]. This finding was reaffirmed by Jonklaas et al., who observed no link between vitamin D levels and thyroid cancer [90]. On assessing a large cohort of 820 patients with PTC, Ahn et al. found that 795 of them had vitamin D insufficiency, but the levels were not related to disease aggressiveness or poor outcome [91]. A recent study of 177 people with papillary thyroid cancer discovered that central adiposity, not vitamin D or adipocytokine status, was the strongest predictor of malignancy [92]. O'Grady et al. studied dietary micronutrient consumption and observed only a correlation between thyroid cancer and low vitamin C intake, but not vitamin D intake [93].

Vitamin D and Hypothyroidism

Only a few research studies have made an attempt to investigate the correlation between serum 25(OH)D levels and hypothyroidism. Mackawy et al. measured serum 25(OH)D levels in 30 hypothyroid patients and 30 healthy participants, discovering that hypothyroid patients had hypovitaminosis D with hypocalcemia, which was signiﬁcantly associated with the degree and severity of hypothyroidism [94]. In a retrospective study done in Turkey, Ucar et al. demonstrated that serum 25(OH)D levels in elderly patients with subclinical hypothyroidism were lower than in healthy controls [95]. Aljohani et al., in a cross-sectional case-control study, found an inverse association between vitamin D status and FT3 [96]. A pilot study in Poland that looked at vitamin D status in the summer months among 133 patients on L-thyroxine treatment showed that vitamin D sufficiency is not achieved even in the summer [97]. Mirhosseini et al. documented that serum 25(OH)D values of more than 125 nmol/L were associated with a 30% decreased risk of hypothyroidism and a 32% reduced risk of elevated anti-thyroid antibodies [98]. Pezeshki et al. conducted a pilot randomized clinical trial in 2020 to investigate the efficacy of vitamin D therapy on subclinical hypothyroidism. The researchers discovered that using vitamin D supplements significantly reduced TSH mean levels, emphasizing the need for screening and vitamin D treatment in subclinical hypothyroid individuals [99]. Musa et al. observed that hypothyroid patients had significantly higher TSH and lower FT4 levels compared to healthy controls in a case-control study of 116 females with hypothyroidism in Saudi Arabia. The authors reported the absence of a noticeable difference in FT3 and 25 (OH)D levels between the two groups [100]. A very recent observational study discovered no association between vitamin D and HT; however, the findings suggested that there may be a subtle decrease in vitamin D levels associated with overt hypothyroidism [101].

Role of supplementation

The soaring data linking vitamin D deficiency to thyroid disorders has piqued researchers' interest in exploring the use of vitamin D supplements in the prevention and treatment of thyroid disorders. An interventional study was conducted on Greek Caucasian patients with HT and hypovitaminosis D to determine if vitamin D3 therapy was effective in the management of these individuals. Four months of cholecalciferol supplementation resulted in a considerable drop (20.3%) in serum anti-TPO levels in these individuals [102]. Chaudhary et al. randomly assigned AITD patients to one of two groups: the first group received cholecalciferol 6000 IU + calcium 500 mg/day for eight weeks, while the second group received only calcium 500 mg/day for eight weeks. Anti-TPO titers fell by more than 25% in 68% in the subjects of the first group but only 44% of the subjects in the second group, though this reduction was significant only in those with TSH ≤ 10 mIU/L [103]. Simsek et al. conducted a randomized control trial of 82 AITD patients in which 46 of them were supplemented with vitamin D 100IU/day for one month, and 36 were not supplemented. The authors observed a reduction in anti-TPO and anti-Tg titers only in the supplementation group [104].

Similarly, in a case-control study, 34 women with HT and normal vitamin D status who had been on levothyroxine for at least six months were divided into two groups based on their preference. For six months, 18 of them were given vitamin D supplements containing 2000 IU of vitamin D per day, whereas the other 16 were not. After six months, a fall in antibody titers (primarily anti-TPO) in response to a rise in 25(OH)D was only statistically significant in those with sub-clinical hypothyroidism, and it was reliant on baseline antibody titers [105]. The authors also demonstrated a significant reduction in anti-TPO titers in non-lactating women with postpartum thyroiditis on vitamin D treatment in a baseline value-dependent manner [106]. In a subsequent study, the same authors attempted to examine the efficacy of selenomethionine and vitamin D in lowering thyroid antibody titers in young drug-naive euthyroid males with AITD. Both selenomethionine and vitamin D reduced anti-TPO and anti-Tg antibody titers, although vitamin D had a greater effect on antibody titers among individuals with baseline deficiency [107]. In a randomized, double-blind placebo-controlled clinical trial involving 42 women with HT disease (aged 18-48 years), subjects were randomly divided into two groups; vitamin D or placebo. For three months, patients in the vitamin D and placebo groups received 50000 IU vitamin D and placebo pearls, respectively. A substantial reduction in anti-Tg antibody and TSH hormone in the Vitamin D group compared to the placebo group (p=0.08) was observed. However, there was no significant reduction in anti-TPO antibodies in the vitamin D group compared to the placebo group (p=0.08) [108]. In patients with AITD, Vondra et al. established a positive correlation between free T4/free T3 ratio and vitamin D deficiency, which disappeared after supplementation with cholecalciferol [109]. Koehler et al., in a retrospective study, reported a larger drop in anti-TPO levels among AITD patients who improved their originally inadequate vitamin D level compared to a control group that maintained vitamin D level below the threshold [110].

A positive association between high dietary vitamin D consumption and thyroid cancer risk was identified in the Vitamins and Lifestyle (VITAL) cross-sectional cohort research (OR=1.66, 95% CI 1.21-2.28) [111]. A systematic review and meta-analysis, consisting of six randomized control trials and 344 AITD patients, Wang et al. concluded that supplementation with vitamin D for six months appears to reduce the thyroid autoantibody titers significantly [112]. Mack et al., in a population-based case-control study centered in Los Angeles, reported that weekly administration of a vitamin D supplement was not linked to the risk of thyroid cancer in females [113]. In a randomized, double-blind study comprising participants with South Asian, Middle Eastern, and African ethnicities with baseline vitamin D insufficiency, Knutsen et al. concluded that low dose vitamin D supplementation did not result in any appreciable improvement in anti-TPO and TSH titers [114]. The impact of vitamin D supplementation may be affected by other variables such as testosterone status. Compared to testosterone-naive males, correction of testosterone levels and vitamin D treatment in testosterone-deficient males has been related to a more dramatic reduction in thyroid autoantibody titers and enhanced thyroid secretory capacity (SPINA-GT index) [115].

Krysiak et al., in a cohort study, suggested the possibility of an additive impact of vitamin D and selenium supplementation on thyroid autoimmunity [116]. In a different study, the authors also addressed the significance of simvastatin in magnifying the effect of vitamin D on thyroid autoimmunity in vitamin D-deficient women with HT [117]. A six-month high-dose atorvastatin therapy was attributed to decreased thyroid antibody titers only in women with normal vitamin D status, suggesting that vitamin D sufficiency indirectly exerts a favorable influence on thyroid autoimmunity [118]. Talaei et al. studied the effects of vitamin D supplementation on thyroid function in hypothyroid patients and reported that 12 weeks of vitamin D supplementation in individuals with primary hypothyroidism was associated with an independent reduction in serum TSH levels [119]. The specific advantages of vitamin D supplementation in thyroid diseases remain elusive, and further randomized controlled studies are required to offer relevant and definite data.

Table 1 gives details of some clinical studies showing the various associations between Vitamin D and thyroid [42,46,48,49,52,59,82,83,84,85,88,90,94,98,99,100,102,103,104,109], all of which are mentioned in this review.

**Table 1:** Clinical studies showing the association between vitamin D and thyroid 25(OH)D: 25-hydroxyvitamin D; anti-TPO: anti-thyroid peroxidase; anti-Tg: anti-thyroglobulin; AITD: autoimmune thyroid disease; HT: Hashimoto’s thyroid disease; GD: Grave’s disease; VDS: vitamin D sufficient; VDD: Vitamin D deficient; PTC: papillary thyroid carcinoma; LMN: lymph node metastasis; ETE: extrathyroidal extension; FTC: follicular thyroid cancer; ATC: anaplastic thyroid cancer; MNG: multinodular nontoxic goiter; TSH: thyroid-stimulating hormone; T4: thyroxine; T3: triiodothyronine

References	Design	Population	Vitamin D deficiency Criteria	Salient Remarks
Goswami et al. 2009 [42]	Community-based survey	642 students, teachers, and staff aged 16-60 years (244 males, 398 females)	<25ng/ml	A weak inverse correlation was found between 25(OH)D and anti-TPO antibody levels (r= -0.08)
Kivity et al. 2011 [46]		50 with AITDs (28 HT, 22 GD), 42 with non-AITDs and 98 healthy subjects	<10ng/ml	Lower levels of vitamin D were documented in 72% AITDs (79% HT and 64% GD), 52% non-AITDs and 30% controls
Tamer et al. 2011 [48]	Case-control study	161 HT patients, 162 healthy controls	<30ng/ml	92% of HT patients and 63% of healthy controls showed vitamin D deficiency
Bozkurt et al. 2013 [49]		180 euthyroid HT, 180 newly diagnosed HT, 180 healthy controls	<25ng/ml	Vitamin D deficiency was demonstrated in 48.3% of euthyroid HT, 35% of newly diagnosed HT, 20.5% of controls. Serum 25(OH)D levels were correlated with thyroid volume (r= 0.15), anti-TPO (r= -0.36), anti-Tg levels (r= -0.34)
Unal et al. 2014 [52]		254 newly diagnosed HT, 27 GD, 124 healthy controls	<20ng/ml	65% of the AITD patients were vitamin D deficient (63% of newly diagnosed HT patients and 85.2% of GD patients). Serum 25(OH)D levels were correlated with anti-TPO (r= -0.18) and anti-Tg levels (r= -0.14)
Evliyaoglu et al. 2015 [59]	Case-control study	90 HT patients (of ages 12.32 ± 2.87 years) and 79 age-matched healthy controls (11.85 ± 2.28 years)	<20ng/ml	71.1% of HT and 51.9% of healthy control had vitamin D deficiency
Roskies et al. 2012 [82]	Retrospective cohort study	212 patients undergoing thyroidectomy	<37.5 nmol/L	The malignancy rate rises when comparing VDS (37.5%) and VDD groups (75%)
Sahin et al. 2013 [83]		344 PTC patients, 116 controls	<20ng/ml	Vitamin D deficiency in 71% PTC, 59% controls. Association between tumour diameter and log-vitamin D3 concentrations (B = 0.207; p = 0.04)
Kim et al. 2014 [84]		548 female PTC patients undergoing total thyroidectomy	18.5ng/ml (median)	Lower vitamin D levels were noted in subjects with tumor size >1cm. Subjects with vitamin D levels below the median were at higher risk of T stage 3/4, LNM, lateral LNM, stage III/IV, and ETE.
Stepien et al. 2010 [85]		50 thyroid cancer patients (27 PTC, 16 FTC, 7 ATC), 34 MNG, and 26 healthy controls		Lower vitamin D levels were observed in thyroid cancer patients than healthy controls. An inverse correlation was noted between vitamin D levels and tumor stage.
Laney et al. 2010 [88]		69 thyroid cancer patients (24 active and 45 in remission), 42 benign thyroid nodule patients	<75nmol/L	No difference was found in vitamin D deficiency rates between the groups
Jonklaas et al. 2013 [90]		65 euthyroid patients undergoing thyroidectomy	<30ng/ml	No associations were identified between vitamin D levels and diagnosis, disease stage, malignancy rate, prognostic features of thyroid cancer.
Mackawy et al. 2013 [94]		30 patients with hypothyroidism and 30 healthy controls	<20ng/ml	Vitamin D was found to be significantly lower in hypothyroid patients than in controls (t= -11.128, P= 0.000)
Pezeshki et al. 2020 [99]	Randomized clinical trial	59 patients diagnosed with both subclinical hypothyroidism and vitamin D deficiency	<30ng/ml	Vitamin D supplementation significantly reduced mean TSH levels (P-value<0.001)
Musa et al. 2017 [100]	Case-control study	58 female patients with hypothyroidism, 58 healthy controls	<30ng/ml	No significant difference was seen in vitamin D levels among females with hypothyroidism and healthy controls.
Mirhosseini et al. 2017 [98]		11,017 participants of a health and wellness program providing vitamin D supplementation to target physiological serum 25(OH)D level above 100nmol/L	<50nmol/L	Hypothyroidism was found in 2% (23% including subclinical hypothyroidism) of participants at baseline and 0.4% (or 6% with subclinical) at follow-up. Optimal thyroid function requires serum 25(OH)D concentration above 125 nmol/L.
Chaudhary et al. 2016 [103]	Open- labeled randomized controlled trial	112 AITD patients	<50nmol/L	Eight weeks of vitamin D supplementation caused a significant reduction in anti-TPO antibody titers.
Mazokopakis et al. 2015 [102]		218 Greek Caucasian HT patients	<30ng/ml	A negative correlation was observed between baseline 25(OH)D and anti-TPO levels. Four months of vitamin D supplementation caused a significant decrease in anti-TPO titers.
Simsek et al. 2016 [104]	Randomized controlled trial	82 AITD patients	<20ng/ml	Reduction in anti-TPO and anti-Tg titers only in the group supplemented with vitamin D 1000 IU/day for one month.
Vondra et al. 2017 [109]		30 female AITD patients	<20ng/ml	A positive relationship was established between the free T4/free T3 ratio and vitamin D deficiency, which disappeared after supplementation with 4300 IU/day of cholecalciferol for three months

Prognostic value

Given that vitamin D deficiency is a curable condition, it is vital to look at its prognostic importance in thyroid disorders. Sulibhavi et al. reviewed 334 patient records for papillary thyroid cancer stage, history of vitamin D deficiency, vitamin D status, and other factors. According to the data, vitamin D deficiency may be advantageous as a negative prognostic indicator in PTC, and preoperative laboratory evaluation may be less useful [120]. Danilovic et al. examined 433 individuals who underwent thyroidectomy for thyroid nodules and found no significant variations in preoperative 25(OH)D levels or quartile distribution between benign and malignant cases. In addition, among 187 individuals with PTC, lower vitamin D levels were not found to be overtly related to a worse prognosis [121]. Kim et al. assessed 410 patients who underwent fine-needle aspiration for thyroid nodules and found that serum 25(OH)D levels and the prevalence of vitamin D insufficiency did not differ substantially between benign and malignant thyroid nodules. Furthermore, vitamin D insufficiency was not linked to disease stage or any other prognostic factors in 44 DTC patients [122]. Choi et al. evaluated 5186 euthyroid individuals with no clinical indications of AITD who had routine health checks and discovered that serum 25(OH)D levels were unrelated to thyroid cancer prevalence [123]. The retrospective study by Kim et al. demonstrated that PTC patients with 25(OH)D levels less than 46.2 nmol/L (median) were at a considerably higher risk of T stage 3/4, lymph node metastasis, and progression to stage III/IV [92]. Lower 1,25(OH)2D3 levels were shown to be inversely related to the advanced tumor stage, according to Stepien et al. (Table 1) [85]. The prognostic role of vitamin D in thyroid disorders has to be widely researched, as it may have the potential to mitigate a disease that is epidemiologically quite common.

Limitations

Hypothyroidism is a multifaceted illness, and we've touched only one component, vitamin D. It is, however, accompanied by a plethora of risk variables, including genetic factors, environmental factors, and autoimmunity. In addition, vitamin D regulates the levels of other hormones such as the parathyroid hormone, although we have not taken this into account. It is beyond the remit of this article to evaluate all of those aspects; therefore, we have focused solely on vitamin D and thyroid.

## Conclusions

The primary objective of this review was to compile the most relevant research in order to better understand the effects of vitamin D in hypothyroidism. The relevance of vitamin D in autoimmune thyroid diseases (AITD), hypothyroidism, and thyroid cancer has received much attention, although the results have been conflicting. The overwhelming amount of evidence pointed to a correlation between vitamin D deficiency and an increased risk of having or developing hypothyroidism, elevated thyroid antibody titers, and thyroid cancer. As per existing research, vitamin D status, receptor polymorphisms, and enzymes involved in its metabolism all play a minor but impactful role in AITD. There seems to be a dynamic relationship between the severity of Hashimoto's disease and vitamin D deficiency. An inverse association was recognized between serum vitamin D concentrations and anti-thyroid antibody levels. Normal physiological vitamin D concentrations appear to be required for optimal thyroid function. Vitamin D supplementation was shown to lower mean TSH levels and anti-TPO levels, and vitamin D deficiency may be beneficial as a negative prognostic indicator.

There is ample literature studying the complex etiology of vitamin D deficiency in thyroid diseases; however, the data is inconclusive. The need to resolve the ambiguity around the causal relationship between vitamin D and thyroid disorders is indispensable, as vitamin D supplementation is inexpensive and has minimal side effects, making it potentially revolutionary in the management of hypothyroidism. We recommend more future studies examining the effects of vitamin D or its analogs in the prevention of AITD and its potential use in the treatment and prognosis of AITD as it would aid in crystallizing the conceptualizations and tailoring the treatment strategy of hypothyroidism with vitamin D.

## References

[1] Aoki Y, Belin RM, Clickner R, Jeffries R, Phillips L, Mahaffey KR (2007). Serum TSH and total T4 in the United States population and their association with participant characteristics: National Health and Nutrition Examination Survey (NHANES 1999-2002). Thyroid.

[2] Garmendia Madariaga A, Santos Palacios S, Guillén-Grima F, Galofré JC (2014). The incidence and prevalence of thyroid dysfunction in Europe: a meta-analysis. J Clin Endocrinol Metab.

[3] Asvold BO, Vatten LJ, Bjøro T (2013). Changes in the prevalence of hypothyroidism: the HUNT Study in Norway. Eur J Endocrinol.

[4] Canaris GJ, Manowitz NR, Mayor G, Ridgway EC (2000). The Colorado thyroid disease prevalence study. Arch Intern Med.

[5] Hollowell JG, Staehling NW, Flanders WD, Hannon WH, Gunter EW, Spencer CA, Braverman LE (2002). Serum TSH, T(4), and thyroid antibodies in the united states population (1988 to 1994): national health and nutrition examination survey (NHANES III). J Clin Endocrinol Metab.

[6] Vanderpump MP (2011). The epidemiology of thyroid disease. Br Med Bull.

[7] Almandoz JP, Gharib H (2012). Hypothyroidism: etiology, diagnosis, and management. Med Clin North Am.

[8] Jonklaas J, Bianco AC, Bauer AJ (2014). Guidelines for the treatment of hypothyroidism: prepared by the American Thyroid Association task force on thyroid hormone replacement. Thyroid.

[9] Gil Á, Plaza-Diaz J, Mesa MD (2018). Vitamin D: classic and novel actions. Ann Nutr Metab.

[10] Kim D (2017). The role of vitamin D in thyroid diseases. Int J Mol Sci.

[11] McDonnell DP, Pike JW, O'Malley BW (1988). The vitamin D receptor: a primitive steroid receptor related to thyroid hormone receptor. J Steroid Biochem.

[12] Lips P (2006). Vitamin D physiology. Prog Biophys Mol Biol.

[13] Holick MF (1987). Photosynthesis of vitamin D in the skin: effect of environmental and life-style variables. Fed Proc.

[14] Chang SW, Lee HC (2019). Vitamin D and health - the missing vitamin in humans. Pediatr Neonatol.

[15] Holick MF (2004). Sunlight and vitamin D for bone health and prevention of autoimmune diseases, cancers, and cardiovascular disease. Am J Clin Nutr.

[16] Holick MF (2004). Vitamin D: importance in the prevention of cancers, type 1 diabetes, heart disease, and osteoporosis. Am J Clin Nutr.

[17] Tangpricha V, Koutkia P, Rieke SM, Chen TC, Perez AA, Holick MF (2003). Fortification of orange juice with vitamin D: a novel approach for enhancing vitamin D nutritional health. Am J Clin Nutr.

[18] Holick MF (2007). Vitamin D deficiency. N Engl J Med.

[19] Holick MF, MacLaughlin JA, Clark MB (1980). Photosynthesis of previtamin D3 in human skin and the physiologic consequences. Science.

[20] Hossein-nezhad A, Holick MF (2012). Optimize dietary intake of vitamin D: an epigenetic perspective. Curr Opin Clin Nutr Metab Care.

[21] Hossein-nezhad A, Holick MF (2013). Vitamin D for health: a global perspective. Mayo Clin Proc.

[22] Lee S, Clark SA, Gill RK, Christakos S (1994). 1,25-dihydroxyvitamin D3 and pancreatic beta-cell function: vitamin D receptors, gene expression, and insulin secretion. Endocrinology.

[23] Tsoukas CD, Provvedini DM, Manolagas SC (1984). 1,25-dihydroxyvitamin D3: a novel immunoregulatory hormone. Science.

[24] Weishaar RE, Simpson RU (1989). The involvement of the endocrine system in regulating cardiovascular function: emphasis on vitamin D3. Endocr Rev.

[25] Sowers JR, Walker S, Sollars E, Herzog J (1980). Inhibition of anterior pituitary hormone release by infusion of calcium chloride in the rat. Endocrinology.

[26] Törnquist K, Lamberg-Allardt C (1987). Systemic effects of 1,25-dihydroxyvitamin D3 on the pituitary-hypothalamic axis in rats. Acta Endocrinol (Copenh).

[27] Lamberg-Allardt C, Valtonen E, Polojärvi M, Stewen P (1991). Characterization of a 1,25-dihydroxy-vitamin d3 receptor in frtl-5 cells. Evidence for an inhibitory effect of 1,25-dihydroxy-vitamin d3 on thyrotropin-induced iodide uptake. Mol Cell Endocrinol.

[28] Berg JP, Liane KM, Bjørhovde SB, Bjøro T, Torjesen PA, Haug E (1994). Vitamin D receptor binding and biological effects of cholecalciferol analogues in rat thyroid cells. J Steroid Biochem Mol Biol.

[29] Egrise D, Burniat A, Lefort A (2010). Vitamin D deficiency modulates the regulation of the pituitary-thyroid axis and thyroid gene expression [Abstract no. 2316].

[30] Clinckspoor I, Verlinden L, Mathieu C, Bouillon R, Verstuyf A, Decallonne B (2013). Vitamin D in thyroid tumorigenesis and development. Prog Histochem Cytochem.

[31] Wang X, Cheng W, Ma Y, Zhu J (2017). Vitamin D receptor gene FokI but not TaqI, ApaI, BsmI polymorphism is associated with Hashimoto's thyroiditis: a meta-analysis. Sci Rep.

[32] Yazici D, Yavuz D, Tarcin O, Sancak S, Deyneli O, Akalin S (2013). Vitamin D receptor gene ApaI, TaqI, FokI and BsmI polymorphisms in a group of Turkish patients with Hashimoto's thyroiditis. Minerva Endocrinol.

[33] Costante G, Durante C, Francis Z, Schlumberger M, Filetti S (2009). Determination of calcitonin levels in C-cell disease: clinical interest and potential pitfalls. Nat Clin Pract Endocrinol Metab.

[34] Pani MA, Regulla K, Segni M (2002). Vitamin D 1alpha-hydroxylase (CYP1alpha) polymorphism in Graves' disease, Hashimoto's thyroiditis and type 1 diabetes mellitus. Eur J Endocrinol.

[35] Wang SH, Koenig RJ, Giordano TJ, Myc A, Thompson NW, Baker JR Jr (1999). 1Alpha,25-dihydroxyvitamin D3 up-regulates Bcl-2 expression and protects normal human thyrocytes from programmed cell death. Endocrinology.

[36] D'Aurizio F, Villalta D, Metus P, Doretto P, Tozzoli R (2015). Is vitamin D a player or not in the pathophysiology of autoimmune thyroid diseases?. Autoimmun Rev.

[37] Baeke F, Takiishi T, Korf H, Gysemans C, Mathieu C (2010). Vitamin D: modulator of the immune system. Curr Opin Pharmacol.

[38] Tokuda N, Mano T, Levy RB (1990). 1,25-Dihydroxyvitamin D3 antagonizes interferon-gamma-induced expression of class II major histocompatibility antigens on thyroid follicular and testicular Leydig cells. Endocrinology.

[39] Fournier C, Gepner P, Sadouk M, Charreire J (1990). In vivo beneficial effects of cyclosporin a and 1,25-dihydroxyvitamin d3 on the induction of experimental autoimmune thyroiditis. Clin Immunol Immunopathol.

[40] Chen W, Lin H, Wang M (2002). Immune intervention effects on the induction of experimental autoimmune thyroiditis. J Huazhong Univ Sci Technolog Med Sci.

[41] Liu S, Xiong F, Liu EM, Zhu M, Lei PY (2010). Effects of 1,25-dihydroxyvitamin D3 in rats with experimental autoimmune thyroiditis [Article in Chinese]. Nan Fang Yi Ke Da Xue Xue Bao.

[42] Goswami R, Marwaha RK, Gupta N (2009). Prevalence of vitamin D deficiency and its relationship with thyroid autoimmunity in Asian Indians: a community-based survey. Br J Nutr.

[43] Chailurkit LO, Aekplakorn W, Ongphiphadhanakul B (2013). High vitamin D status in younger individuals is associated with low circulating thyrotropin. Thyroid.

[44] Zhang Q, Wang Z, Sun M (2014). Association of high vitamin d status with low circulating thyroid-stimulating hormone independent of thyroid hormone levels in middle-aged and elderly males. Int J Endocrinol.

[45] Effraimidis G, Badenhoop K, Tijssen JG, Wiersinga WM (2012). Vitamin D deficiency is not associated with early stages of thyroid autoimmunity. Eur J Endocrinol.

[46] Kivity S, Agmon-Levin N, Zisappl M (2011). Vitamin D and autoimmune thyroid diseases. Cell Mol Immunol.

[47] Shin DY, Kim KJ, Kim D, Hwang S, Lee EJ (2014). Low serum vitamin D is associated with anti-thyroid peroxidase antibody in autoimmune thyroiditis. Yonsei Med J.

[48] Tamer G, Arik S, Tamer I, Coksert D (2011). Relative vitamin D insufficiency in Hashimoto's thyroiditis. Thyroid.

[49] Bozkurt NC, Karbek B, Ucan B, Sahin M, Cakal E, Ozbek M, Delibasi T (2013). The association between severity of vitamin D deficiency and Hashimoto's thyroiditis. Endocr Pract.

[50] Choi YM, Kim WG, Kim TY (2014). Low levels of serum vitamin D3 are associated with autoimmune thyroid disease in pre-menopausal women. Thyroid.

[51] Yasuda T, Okamoto Y, Hamada N (2012). Serum vitamin D levels are decreased and associated with thyroid volume in female patients with newly onset Graves' disease. Endocrine.

[52] Unal AD, Tarcin O, Parildar H, Cigerli O, Eroglu H, Demirag NG (2014). Vitamin D deficiency is related to thyroid antibodies in autoimmune thyroiditis. Cent Eur J Immunol.

[53] Rotondi M, Chiovato L (2013). Vitamin D deficiency in patients with Graves' disease: probably something more than a casual association. Endocrine.

[54] Ma J, Wu D, Li C (2015). Lower serum 25-hydroxyvitamin D level is associated with 3 types of autoimmune thyroid diseases. Medicine (Baltimore).

[55] Ke W, Sun T, Zhang Y, He L, Wu Q, Liu J, Zha B (2017). 25-Hydroxyvitamin D serum level in Hashimoto's thyroiditis, but not Graves' disease is relatively deficient. Endocr J.

[56] Camurdan OM, Döğer E, Bideci A, Celik N, Cinaz P (2012). Vitamin D status in children with Hashimoto thyroiditis. J Pediatr Endocrinol Metab.

[57] Metwalley KA, Farghaly HS, Sherief T, Hussein A (2016). Vitamin D status in children and adolescents with autoimmune thyroiditis. J Endocrinol Invest.

[58] Sönmezgöz E, Ozer S, Yilmaz R, Önder Y, Bütün I, Bilge S (2016). Hypovitaminosis D in children with Hashimoto's thyroiditis [Article in Spanish]. Rev Med Chil.

[59] Evliyaoğlu O, Acar M, Özcabı B, Erginöz E, Bucak F, Ercan O, Kucur M (2015). Vitamin D deficiency and Hashimoto's thyroiditis in children and adolescents: a critical vitamin D level for this association?. J Clin Res Pediatr Endocrinol.

[60] Răcătăianu N, Leach NV, Bolboacă SD (2018). Vitamin D deficiency, insulin resistance and thyroid dysfunction in obese patients: is inflammation the common link?. Scand J Clin Lab Invest.

[61] Yasmeh J, Farpour F, Rizzo V, Kheradnam S, Sachmechi I (2016). Hashimoto thyroiditis not associated with vitamin D deficiency. Endocr Pract.

[62] Bizzaro G, Shoenfeld Y (2015). Vitamin D and thyroid autoimmune diseases: the known and the obscure. Immunol Res.

[63] Galesanu C, Mocanu V (2015). Vitamin D deficiency and the clinical consequences. Rev Med Chir Soc Med Nat Iasi.

[64] Kawicka A, Regulska-Ilow B, Regulska-Ilow B (2015). Metabolic disorders and nutritional status in autoimmune thyroid diseases. Postepy Hig Med Dosw (Online).

[65] Walker MD, Zylberberg HM, Green PH, Katz MS (2019). Endocrine complications of celiac disease: a case report and review of the literature. Endocr Res.

[66] Krysiak R, Szkróbka W, Okopień B (2019). The effect of gluten-free diet on thyroid autoimmunity in drug-naïve women with hashimoto's thyroiditis: a pilot study. Exp Clin Endocrinol Diabetes.

[67] Waterhouse JC, Perez TH, Albert PJ (2009). Reversing bacteria-induced vitamin D receptor dysfunction is key to autoimmune disease. Ann N Y Acad Sci.

[68] Giovannucci E (2009). Vitamin D and cancer incidence in the Harvard cohorts. Ann Epidemiol.

[69] Dackiw AP, Ezzat S, Huang P, Liu W, Asa SL (2004). Vitamin D3 administration induces nuclear p27 accumulation, restores differentiation, and reduces tumor burden in a mouse model of metastatic follicular thyroid cancer. Endocrinology.

[70] Zabel M, Flig K, Gebarowska E, Surdyk-Zasada J (2003). The effect of calcitriol and its analogues on proliferation and hormone expression in cultured cells of thyroid medullary carcinomas. Folia Morphol (Warsz).

[71] Zabel M, Gebarowska E, Drag-Zalesińska M, Wysocka T (2002). Effect of calcitriol on proliferation of TT cells and on expression of calcitonin gene. Folia Histochem Cytobiol.

[72] Liu W, Asa SL, Ezzat S (2005). 1alpha,25-Dihydroxyvitamin D3 targets PTEN-dependent fibronectin expression to restore thyroid cancer cell adhesiveness. Mol Endocrinol.

[73] Khadzkou K, Buchwald P, Westin G, Dralle H, Akerström G, Hellman P (2006). 25-hydroxyvitamin D3 1alpha-hydroxylase and vitamin D receptor expression in papillary thyroid carcinoma. J Histochem Cytochem.

[74] Izkhakov E, Somjen D, Sharon O (2016). Vitamin D receptor expression is linked to potential markers of human thyroid papillary carcinoma. J Steroid Biochem Mol Biol.

[75] Clinckspoor I, Hauben E, Verlinden L (2012). Altered expression of key players in vitamin D metabolism and signaling in malignant and benign thyroid tumors. J Histochem Cytochem.

[76] Chiang KC, Kuo SF, Chen CH (2015). MART-10, the vitamin D analog, is a potent drug to inhibit anaplastic thyroid cancer cell metastatic potential. Cancer Lett.

[77] Somjen D, Grafi-Cohen M, Posner GH, Sharon O, Kraiem Z, Stern N (2013). Vitamin D less-calcemic analog modulates the expression of estrogen receptors, vitamin D receptor and 1α-hydroxylase 25-hydroxy vitamin D in human thyroid cancer cell lines. J Steroid Biochem Mol Biol.

[78] Clinckspoor I, Verlinden L, Overbergh L (2011). 1,25-dihydroxyvitamin D3 and a superagonistic analog in combination with paclitaxel or suberoylanilide hydroxamic acid have potent antiproliferative effects on anaplastic thyroid cancer. J Steroid Biochem Mol Biol.

[79] Suzuki S, Takenoshita S, Furukawa H, Tsuchiya A (1999). Antineoplastic activity of 1,25(OH)2D3 and its analogue 22-oxacalcitriol against human anaplastic thyroid carcinoma cell lines in vitro. Int J Mol Med.

[80] Liu W, Asa SL, Fantus IG, Walfish PG, Ezzat S (2002). Vitamin D arrests thyroid carcinoma cell growth and induces p27 dephosphorylation and accumulation through PTEN/akt-dependent and -independent pathways. Am J Pathol.

[81] Bennett RG, Wakeley SE, Hamel FG, High RR, Korch C, Goldner WS (2012). Gene expression of vitamin D metabolic enzymes at baseline and in response to vitamin D treatment in thyroid cancer cell lines. Oncology.

[82] Roskies M, Dolev Y, Caglar D, Hier MP, Mlynarek A, Majdan A, Payne RJ (2012). Vitamin D deficiency as a potentially modifiable risk factor for thyroid cancer. J Otolaryngol Head Neck Surg.

[83] Sahin M, Uçan B, Giniş Z (2013). Vitamin D3 levels and insulin resistance in papillary thyroid cancer patients. Med Oncol.

[84] Kim JR, Kim BH, Kim SM (2014). Low serum 25 hydroxyvitamin D is associated with poor clinicopathologic characteristics in female patients with papillary thyroid cancer. Thyroid.

[85] Stepien T, Krupinski R, Sopinski J, Kuzdak K, Komorowski J, Lawnicka H, Stepien H (2010). Decreased 1-25 dihydroxyvitamin D3 concentration in peripheral blood serum of patients with thyroid cancer. Arch Med Res.

[86] Penna-Martinez M, Ramos-Lopez E, Stern J (2009). Vitamin D receptor polymorphisms in differentiated thyroid carcinoma. Thyroid.

[87] Penna-Martinez M, Ramos-Lopez E, Stern J (2012). Impaired vitamin D activation and association with CYP24A1 haplotypes in differentiated thyroid carcinoma. Thyroid.

[88] Laney N, Meza J, Lyden E, Erickson J, Treude K, Goldner W (2010). The prevalence of vitamin D deficiency is similar between thyroid nodule and thyroid cancer patients. Int J Endocrinol.

[89] Lizis-Kolus K, Hubalewska-Dydejczyk A, Trofimiuk-Muldnerz M, Sowa-Staszczak A, Kowalska A (2013). Assessment of 25(OH)D3, concentration levels in patients with papillary thyroid cancer compared to patients with Hashimoto's thyroiditis [Article in Polish]. Przegl Lek.

[90] Jonklaas J, Danielsen M, Wang H (2013). A pilot study of serum selenium, vitamin D, and thyrotropin concentrations in patients with thyroid cancer. Thyroid.

[91] Ahn HY, Chung YJ, Park KY, Cho BY (2016). Serum 25-hydroxyvitamin D level does not affect the aggressiveness and prognosis of papillary thyroid cancer. Thyroid.

[92] Warakomski J, Romuk E, Jarząb B, Krajewska J, Siemińska L (2018). Concentrations of selected adipokines, interleukin-6, and vitamin D in patients with papillary thyroid carcinoma in respect to thyroid cancer stages. Int J Endocrinol.

[93] O'Grady TJ, Kitahara CM, DiRienzo AG, Gates MA (2014). The association between selenium and other micronutrients and thyroid cancer incidence in the NIH-AARP Diet and Health Study. PLoS One.

[94] Mackawy AM, Al-Ayed BM, Al-Rashidi BM (2013). Vitamin D deficiency and its association with thyroid disease. Int J Health Sci (Qassim).

[95] Ucar F, Akyol S, Ozturk G (2014). Evaluation of serum vitamin D levels in elderly patients with subclinical hypothyroidism. J Exper Clin Med.

[96] Aljohani NJ, Al-Daghri NM, Al-Attas OS (2013). Differences and associations of metabolic and vitamin D status among patients with and without sub-clinical hypothyroid dysfunction. BMC Endocr Disord.

[97] Kmieć P, Minkiewicz I, Rola R, Sworczak K, Żmijewski MA, Kowalski K (2018). Vitamin D status including 3-epi-25(OH)D3 among adult patients with thyroid disorders during summer months. Endokrynol Pol.

[98] Mirhosseini N, Brunel L, Muscogiuri G, Kimball S (2017). Physiological serum 25-hydroxyvitamin D concentrations are associated with improved thyroid function-observations from a community-based program. Endocrine.

[99] Pezeshki B, Ahmadi A, Karimi A (2020). The effect of vitamin D replacement on patient [sic] with subclinical hypothyroidism: a pilot randomized clinical trial. Galen Med J.

[100] Musa IR, Gasim GI, Khan S, Ibrahim IA, Abo-Alazm H, Adam I (2017). No association between 25 (OH) vitamin D level and hypothyroidism among females. Open Access Maced J Med Sci.

[101] Cvek M, Kaličanin D, Barić A (2021). Vitamin D and Hashimoto's thyroiditis: observations from CROHT biobank. Nutrients.

[102] Mazokopakis EE, Papadomanolaki MG, Tsekouras KC, Evangelopoulos AD, Kotsiris DA, Tzortzinis AA (2015). Is vitamin D related to pathogenesis and treatment of Hashimoto's thyroiditis?. Hell J Nucl Med.

[103] Chaudhary S, Dutta D, Kumar M, Saha S, Mondal SA, Kumar A, Mukhopadhyay S (2016). Vitamin D supplementation reduces thyroid peroxidase antibody levels in patients with autoimmune thyroid disease: an open-labeled randomized controlled trial. Indian J Endocrinol Metab.

[104] Simsek Y, Cakır I, Yetmis M, Dizdar OS, Baspinar O, Gokay F (2016). Effects of vitamin D treatment on thyroid autoimmunity. J Res Med Sci.

[105] Krysiak R, Szkróbka W, Okopień B (2017). The effect of vitamin D on thyroid autoimmunity in levothyroxine-treated women with Hashimoto's thyroiditis and normal vitamin D status. Exp Clin Endocrinol Diabetes.

[106] Krysiak R, Kowalcze K, Okopien B (2016). The effect of vitamin D on thyroid autoimmunity in non-lactating women with postpartum thyroiditis. Eur J Clin Nutr.

[107] Krysiak R, Szkróbka W, Okopień B (2019). The effect of vitamin D and selenomethionine on thyroid antibody titers, hypothalamic-pituitary-thyroid axis activity and thyroid function tests in men with Hashimoto's thyroiditis: a pilot study. Pharmacol Rep.

[108] Chahardoli R, Saboor-Yaraghi AA, Amouzegar A, Khalili D, Vakili AZ, Azizi F (2019). Can supplementation with vitamin D modify thyroid autoantibodies (anti-TPO Ab, anti-Tg Ab) and thyroid profile (T3, T4, TSH) in Hashimoto's thyroiditis? A double blind, randomized clinical trial. Horm Metab Res.

[109] Vondra K, Bílek R, Matucha P, Salátová M, Vosátková M, Stárka L, Hampl R (2017). Vitamin D supplementation changed relationships, not levels of metabolic-hormonal parameters in autoimmune thyroiditis. Physiol Res.

[110] Koehler VF, Filmann N, Mann WA (2019). Vitamin D status and thyroid autoantibodies in autoimmune thyroiditis. Horm Metab Res.

[111] Greenlee H, White E, Patterson RE, Kristal AR (2004). Supplement use among cancer survivors in the vitamins and lifestyle (VITAL) study cohort. J Altern Complement Med.

[112] Wang S, Wu Y, Zuo Z, Zhao Y, Wang K (2018). The effect of vitamin D supplementation on thyroid autoantibody levels in the treatment of autoimmune thyroiditis: a systematic review and a meta-analysis. Endocrine.

[113] Mack WJ, Preston-Martin S, Bernstein L, Qian D (2002). Lifestyle and other risk factors for thyroid cancer in Los Angeles county females. Ann Epidemiol.

[114] Knutsen KV, Madar AA, Brekke M, Meyer HE, Eggemoen ÅR, Mdala I, Lagerløv P (2017). Effect of vitamin D on thyroid autoimmunity: a randomized, double-blind, controlled trial among ethnic minorities. J Endocr Soc.

[115] Krysiak R, Kowalcze K, Okopień B (2019). The effect of vitamin D on thyroid autoimmunity in euthyroid men with autoimmune thyroiditis and testosterone deficiency. Pharmacol Rep.

[116] Krysiak R, Kowalcze K, Okopień B (2019). Selenomethionine potentiates the impact of vitamin D on thyroid autoimmunity in euthyroid women with Hashimoto's thyroiditis and low vitamin D status. Pharmacol Rep.

[117] Krysiak R, Szkróbka W, Okopień B (2018). Moderate-dose simvastatin therapy potentiates the effect of vitamin D on thyroid autoimmunity in levothyroxine-treated women with Hashimoto's thyroiditis and vitamin D insufficiency. Pharmacol Rep.

[118] Krysiak R, Szkróbka W, Okopień B (2019). The relationship between statin action on thyroid autoimmunity and vitamin D status: a pilot study. Exp Clin Endocrinol Diabetes.

[119] Talaei A, Ghorbani F, Asemi Z (2018). The effects of vitamin D supplementation on thyroid function in hypothyroid patients: a randomized, double-blind, placebo-controlled trial. Indian J Endocrinol Metab.

[120] Sulibhavi A, Rohlfing ML, Jalisi SM, McAneny DB, Doherty GM, Holick MF, Noordzij JP (2019). Vitamin D deficiency and its relationship to cancer stage in patients who underwent thyroidectomy for papillary thyroid carcinoma. Am J Otolaryngol.

[121] Danilovic DL, Ferraz-de-Souza B, Fabri AW (2016). 25-hydroxyvitamin d and TSH as risk factors or prognostic markers in thyroid carcinoma. PLoS One.

[122] Kim D (2016). Low vitamin D status is not associated with thyroid cancer risk. J Clin Endocrinol Metab.

[123] Choi YM, Kim WG, Kim TY (2017). Serum vitamin D3 levels are not associated with thyroid cancer prevalence in euthyroid subjects without autoimmune thyroid disease. Korean J Intern Med.

